# Supplementary figures and images for: Incomplete bunyavirus particles can cooperatively support virus infection and spread
Source: PLoS Biol. 2022 Nov 15;20(11):e3001870. doi: 10.1371/journal.pbio.3001870 (PMC9665397; doi:10.1371/journal.pbio.3001870)

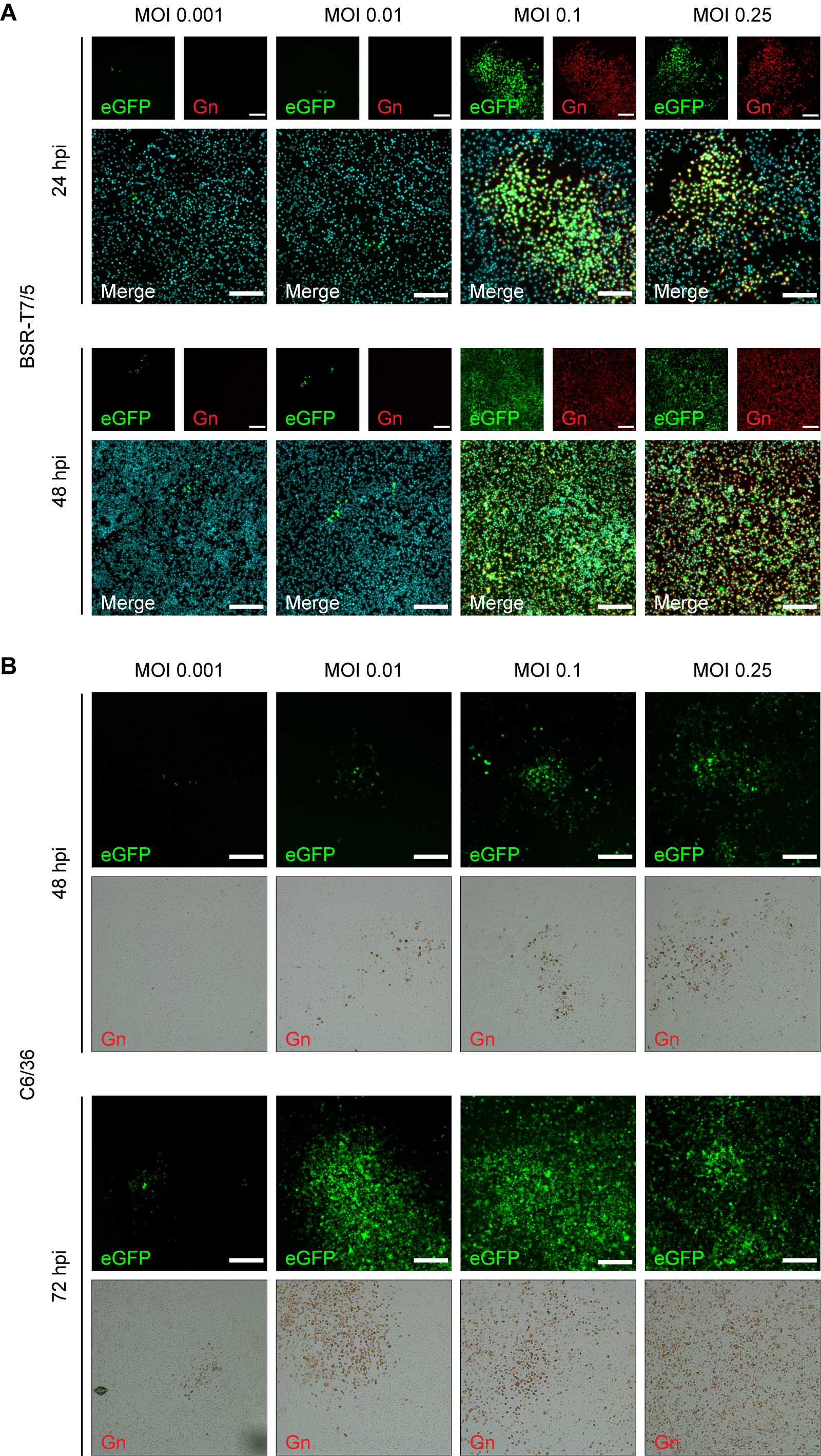

Supplement: S1 Fig — (A and B) Mammalian (BSR-T7/5) (A) and insect (C6/36) (B) cells were simultaneously infected with non-spreading iRVFV-SL-eGFP and iRVFV-ML particles at increasing MOIs (ranging from 0.001 to 0.25 for each virus). Co-infection with the two populations of incomplete RVFV particles supports genome complementation, allows virus replication, production of infectious progeny, and virus spread. Infected cells were analyzed at 24–48 h (BSR-T7/5 cells) or 48–72 h (C6/36 cells) post-infection by following the expression of eGFP (green) via direct fluorescence microscopy examination and the expression of Gn (red) via an immunofluorescence assay in BSR-T7/5 cells or an immunoperoxidase monolayer assay in C6/36 cells. Expression of Gn was detected with rabbit polyclonal anti-Gn serum in combination with Alexa Fluor 568–conjugated secondary antibodies (immunofluorescence assay) or with HRP-conjugated secondary antibodies (immunoperoxidase monolayer assay). Cell nuclei (cyan) were visualized with DAPI. Of note, with the sole intention of depicting the outcome progression at increasing MOIs, images corresponding to co-infections (MOI of 0.1) at 24 h (BSR-T/5) and 72 h (C6/36) post-infection were purposely selected to be the exact same images as shown in Fig 4B and 4C. Scale bars, 200 μm. (TIF) [file pbio.3001870.s001.tif]

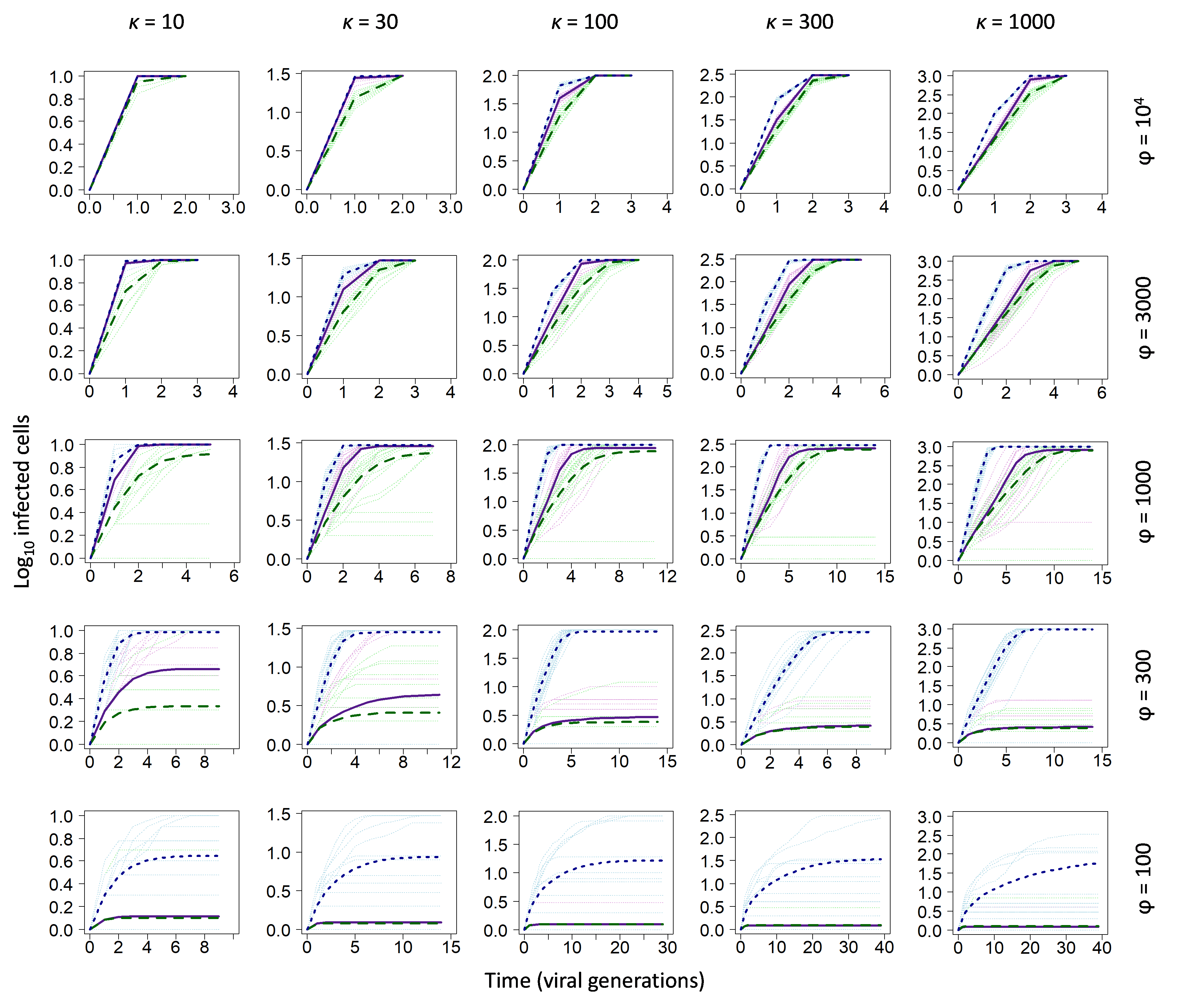

Supplement: S2 Fig — Predicted RVFV infection dynamics in mammalian cells for three different scenarios: selective genome packaging (finely dotted blue lines), non-selective genome packaging without co-infection by incomplete particles (coarsely dotted green lines), and non-selective genome packaging with co-infection and productive complementation by incomplete particles (solid magenta lines). The darker lines (each color corresponding to a different scenario) represent the mean values based on 1,000 simulations. The faint dotted lines (each color corresponding to a different scenario) represent trajectories for 20 individual simulations. In all panels, time is on the x axis and the number of infected cells is on the y axis. Note that the axes are scaled differently over panels. Values for the total number of cells (κ) and virus particle production (φ) were varied over panels, as indicated by the values at the top and right of the figure, respectively. Infection dynamics are clearly affected by parameter values, but under many conditions, the virus employing non-selective packaging but that allows co-infection has an advantage over the virus employing non-selective packaging without co-infection. The code required to reproduce the plots of this figure is provided as S3 File. (PNG) [file pbio.3001870.s002.png]

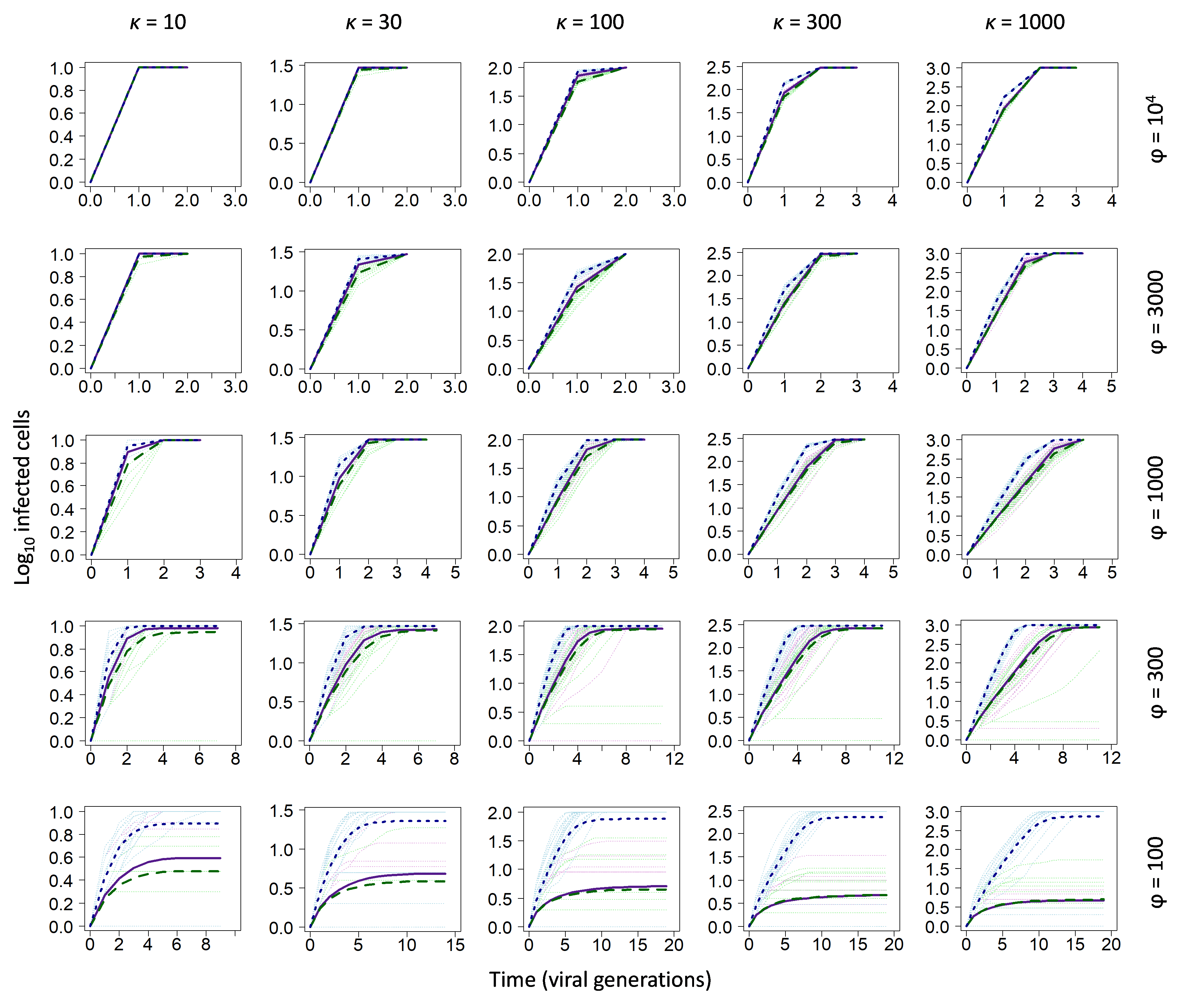

Supplement: S3 Fig — Predicted RVFV infection dynamics in insect cells for three different scenarios: selective genome packaging (finely dotted blue lines), non-selective genome packaging without co-infection by incomplete particles (coarsely dotted green lines), and non-selective genome packaging with co-infection and productive complementation by incomplete particles (solid magenta lines). The darker lines (each color corresponding to a different scenario) represent the mean values based on 1,000 simulations. The faint dotted lines (each color corresponding to a different scenario) represent trajectories for 20 individual simulations. In all panels, time is on the x axis and the number of infected cells is on the y axis. Note that the axes are scaled differently over panels. Values for the total number of cells (κ) and virus particle production (φ) were varied over panels, as indicated by the values at the top and right of the figure, respectively. Infection dynamics are clearly affected by parameter values, but the virus employing non-selective packaging with co-infection generally has a similar performance to the virus employing non-selective packaging without co-infection. The code required to reproduce the plots of this figure is provided as S3 File. (PNG) [file pbio.3001870.s003.png]

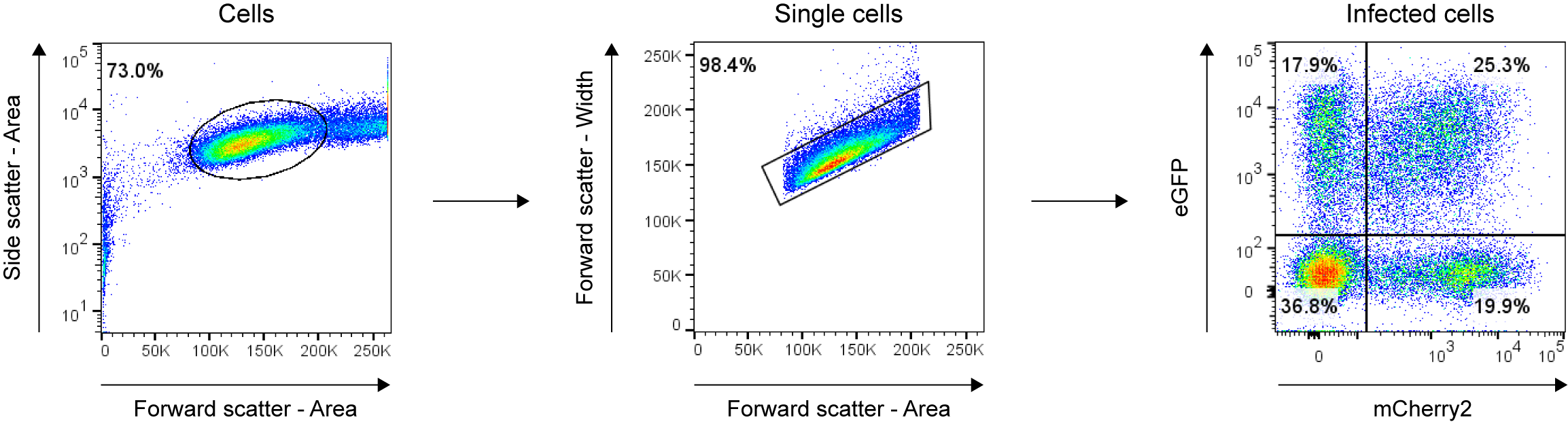

Supplement: S4 Fig — Illustrative example of the gating strategy employed for the analysis of flow cytometry data. BSR-T7/5 cells were mock-infected, singly-infected, or co-infected with non-spreading iRVFV-SL-eGFP and/or iRVFV-SL-mCherry2 particles. The cell population of interest was first discriminated from debris. Then, a gate was applied to select single cell events from doublets. Finally, we quantified the fraction of non-infected, singly-infected, or co-infected cells by determining the expression of eGFP, mCherry2, or both. The plots depicted here correspond to the co-infected sample (MOI of 0.5 for each virus population), as shown in Fig 2F. (TIF) [file pbio.3001870.s004.tif]
