# Supplementary material for: Incomplete bunyavirus particles can cooperatively support virus infection and spread
Source: PLoS Biol. 2022 Nov 15;20(11):e3001870. doi: 10.1371/journal.pbio.3001870 (PMC9665397; doi:10.1371/journal.pbio.3001870)
Supplement: S1 Table — (DOCX) [file pbio.3001870.s005.docx]

**S1 Table. Model parameter estimates and model selection results for the co‑infection assays.**

| Model^a^ | Model parameter estimates^b^ | NLL^c^ | AIC | ΔAIC | AW |
| --- | --- | --- | --- | --- | --- |
| A | - | 10,954.69 | 21,909.38 | 16,984.37 | 0.000 |
| B | *p* = 0.03 | 8,891.86 | 17,785.72 | 12,860.71 | 0.000 |
| C | *p* = 0.95, *α* = 0.13, *β* = 0.83 | 2,459.51 | 4,925.01 | - | 1.000 |

NLL: negative log likehood. AIC: Akaike information criterion. ΔAIC: delta AIC, the difference between this model and the best supported model in AIC. AW: Akaike weight, the likelihood that this model is the best-supported model within the set of models considered. ^a^ For a full description of the models, see the **Materials and methods** section and **S1 File**. ^b^ Note that model A has no free parameters that need to be estimated. ^c^ For a clarification of all terms used here, see this review [1].

**Supporting References**

1. Johnson JB, Omland KS. Model selection in ecology and evolution. Trends in Ecology & Evolution. 2004;19: 101–108. doi:10.1016/j.tree.2003.10.013
