# Supplementary material for: Incomplete bunyavirus particles can cooperatively support virus infection and spread
Source: PLoS Biol. 2022 Nov 15;20(11):e3001870. doi: 10.1371/journal.pbio.3001870 (PMC9665397; doi:10.1371/journal.pbio.3001870)
Supplement: S2 Table — (DOCX) [file pbio.3001870.s006.docx]

**S2 Table.** **Primers for cDNA synthesis of viral genome segments.**

| Target | Name | Sequence |
| --- | --- | --- |
| RVFV-Clone 13-S and RVFV-35/74-S | JR860-For | ACAAAGCTCCCTAGAGATACA |
| RVFV-Clone 13-M and RVFV-35/74-M | JR861-For | GACACAAAGACGGTGCATTA |
| RVFV-Clone 13-L and RVFV-35/74-L | JR890-For | GACACAAAGGCGCCCAATC |
