# Supplementary material for: Incomplete bunyavirus particles can cooperatively support virus infection and spread
Source: PLoS Biol. 2022 Nov 15;20(11):e3001870. doi: 10.1371/journal.pbio.3001870 (PMC9665397; doi:10.1371/journal.pbio.3001870)
Supplement: S3 Table — (DOCX) [file pbio.3001870.s007.docx]

**S3 Table.** **Primers for RT-qPCR amplifications of viral genome fragments and *ampR*.**

| Target | Name | Sequence |
| --- | --- | --- |
| RVFV-Clone 13-S and RVFV-35/74-S | JR907-For | TCCAGTTTGCTGCTCAA |
|  | JR908-Rev | CTGCTTTAAGAGTTCGATAACC |
| RVFV-Clone 13-M and RVFV-35/74-M | JR909-For | GCTGATGGCTTGAACAAC |
|  | JR910-Rev | GTCTCTCACACCGAACTATC |
| RVFV-Clone 13-L and RVFV-35/74-L | JR911-For | TCGATAGATGTGGAAGATATGG |
|  | JR912-Rev | CGTCATTCATCATGGGAAAC |
| *ampR* | JR971-For | GCAGTGTTATCACTCATGG |
|  | JR972-Rev | CACTATTCTCAGAATGACTTGG |
