# Supplementary material for: Incomplete bunyavirus particles can cooperatively support virus infection and spread
Source: PLoS Biol. 2022 Nov 15;20(11):e3001870. doi: 10.1371/journal.pbio.3001870 (PMC9665397; doi:10.1371/journal.pbio.3001870)
Supplement: S4 Table — (DOCX) [file pbio.3001870.s008.docx]

**S4 Table. Antibodies used in immunostaining assays.**

| Assay | Target | Antibody | Dilution | Source/reference |
| --- | --- | --- | --- | --- |
| IPMA, IF (1^ary^) | RVFV Gn | Rabbit polyclonal serum | 1:500 | Thermo Fisher |
| IPMA (2^ary^) | Rabbit IgG | Goat polyclonal anti-rabbit IgG HRP‑conjugated | 1:500 | P0448 Dako |
| IF (2^ary^) | Rabbit IgG | Goat polyclonal anti-rabbit IgG-FITC | 1:250 | sc-2012 Santa Cruz Biotechnology |
| IF (2^ary^) | Rabbit IgG | Donkey polyclonal anti-rabbit IgG-Alexa Fluor 568 | 1:500 | A10042 Invitrogen |
| IF (1^ary^) | RVFV N | Monoclonal mouse hybridoma | 1:100 | F1D11 CISA‑INIA |
| IF (2^ary^) | Mouse IgG | Goat polyclonal anti-mouse IgG-Alexa Fluor Plus 488 | 1:500 | A32723 Invitrogen |
| FISH-IF (1^ary^) | RVFV Gn | Hybridoma 4-D4 supernatant | 1:160 | [1] |
| FISH-IF (2^ary^) | Mouse IgG | Goat polyclonal anti-mouse IgG-Alexa Fluor 488 | 1:1000 | A-11001 Invitrogen |

IPMA: immunoperoxidase monolayer assay, IF: immunofluorescence, FISH-IF: fluorescence *in situ* hybridization-immunofluorescence.

**Supporting References**

1. Keegan K, Collett MS. Use of bacterial expression cloning to define the amino acid sequences of antigenic determinants on the G2 glycoprotein of Rift Valley fever virus. Journal of Virology. 1986;58: 263–270.
