# Supplementary material for: Incomplete bunyavirus particles can cooperatively support virus infection and spread
Source: PLoS Biol. 2022 Nov 15;20(11):e3001870. doi: 10.1371/journal.pbio.3001870 (PMC9665397; doi:10.1371/journal.pbio.3001870)
Supplement: S1 Text — (PDF) [file pbio.3001870.s010.pdf]

Supporting Information

Bermúdez-Méndez E, Bronsvoort KF, Zwart MP, van de Water S, Cárdenas-Rey I, Vloet RPM, Koenraadt CJM, Pijlman GP, Kortekaas J, Wichgers Schreur PJ. (2022) Incomplete bunyavirus particles can cooperatively support virus infection and spread. PLOS Biology.

S1 Text. Sensitivity analysis of the infection model parameters.

We generated an infection model to predict the infection kinetics of RVFV under three different scenarios and for two distributions of genome segments over virus particles (see Fig 7A, Materials and methods, and S3 File). However, the models used have free parameters, so it is relevant to consider how parameter values affect model predictions. The infection model we have generated is relatively simple and has only three free parameters: the total number of cells (κ), virus particle production (φ) and the probability that a virus particle will infect a cell (ρ). As argued in the results section, we expect that parameter values will affect the prediction because the total number of cells available will determine how the multiplicity of infection (MOI) changes over time, i.e. all other things equal, the MOI will increase more rapidly in a small population of cells. It therefore makes sense to consider systematically how κ affects model predictions. We also expect that both φ and ρ will affect the model prediction, as they will determine how efficiently infection spreads between cells. However, the product $\varphi\rho$ determines the mean of the Poisson distribution describing the MOI, so it suffices to consider only one of these two parameters in this analysis, and we have chosen φ. Note that the model has two additional parameters: the total rounds of viral replication (g_{max}) and the number of infected cells in generation zero (i_0). Parameter g_{max} will not affect the prediction, provided that sufficient generations are given for all cells to become infected, and $i_0 = 1$, so that we can explore infection dynamics over the largest range in the frequency of infected cells and MOI values. In sum, to get an indication of how sensitive model predictions are to the chosen model parameters, we only need to consider parameters κ and φ.

We settled on values of $\kappa = 100$ and $\varphi = 1000$ *a priori* as reasonable values for these two parameters. Here, we therefore considered model predictions for all combinations of values of these parameters over two orders of magnitude to gauge how these model parameter values affect infection dynamics: $\kappa = \{10, 30, 100, 300, 1000\}$ and $\varphi = \{100, 300, 1000, 3000, 10^4\}$. In particular, we are interested in how quickly the number of infected cells increases in a virus employing non-selective genome packaging but that allows co-infection compared to a virus employing non-selective packaging without co-infection.

Supporting Information

Bermúdez-Méndez E, Bronsvoort KF, Zwart MP, van de Water S, Cárdenas-Rey I, Vloet RPM, Koenraadt CJM, Pijlman GP, Kortekaas J, Wichgers Schreur PJ. (2022) Incomplete bunyavirus particles can cooperatively support virus infection and spread. PLOS Biology.

First, we considered the predictions for mammalian cells (S2 Fig). Here we found that the virus employing non-selective packaging with co-infection outperformed the virus employing non-selective packaging without co-infection under most conditions. This effect was not seen when virus particle production was very low (φ = 100, both viruses with non-selective packaging perform very poorly) or very high (φ =10^4, both viruses perform so well that differences become negligible). For some conditions (e.g., κ = 10, φ = 300), the performance of the virus employing non-selective packaging with co-infection is much better than the virus employing non-selective packaging without co-infection. In some cases (e.g., κ = 10, φ = 1000), the performance of the virus employing non-selective packaging with co-infection is much closer to that of the virus employing selective packaging than to the virus employing non-selective packaging without co-infection. These results therefore clearly demonstrate that, for mammalian cells, the virus employing non-selective packaging with co-infection is predicted to have an advantage in spread over the virus employing non-selective packaging without co-infection under many conditions.

Second, we considered the predictions for insect cells (S3 Fig), which differ only from the mammalian cell scenario in the distribution of genome segments over virus particles assumed. Recall that the model did not predict a large difference for the parameters values chosen (Fig 7A). Here we found a similar pattern, as performance was the same or only marginally better for the virus that benefits from co-infection under most conditions. The advantage of the co-infecting virus over the non-co-infecting virus was greatest at low number of cells ($\kappa \leq 30$) and low virus particle production ($\varphi \leq 300$). Overall, these results suggest that the distribution of genome segments over virus particles may have a larger effect on whether co-infection contributes to enhanced spread than the exact conditions (κ and φ values) under which the virus is replicating.
