# Supplementary material for: Incomplete bunyavirus particles can cooperatively support virus infection and spread
Source: PLoS Biol. 2022 Nov 15;20(11):e3001870. doi: 10.1371/journal.pbio.3001870 (PMC9665397; doi:10.1371/journal.pbio.3001870)
Supplement: S3 File — (HTML) [file pbio.3001870.s013.html]

S3 File. Modeling virus spread: simple sensitivity analysis


# S3 File. Modeling virus spread: simple sensitivity analysis

#### Author: Mark P. Zwart

### Supporting Information

#### Bermúdez-Méndez E, Bronsvoort KF, Zwart MP, van de Water S, Cárdenas-Rey I, Vloet RPM, Koenraadt CJM, Pijlman GP, Kortekaas J, Wichgers Schreur PJ. (2022) Incomplete bunyavirus particles can cooperatively support virus infection and spread. PLOS Biology.

#### Intro

**S3 File** is a simplified version of **S2
File** to explore the effects of model parameter values on
predictions as presented in **S2** and **S3
Figs**. This version of the code only predicts the number of
infected cells over time, and does not consider the relationship between
MOI and infections by incomplete particles.

The model parameters are set in the section “General conditions for
simulations”. To generate the different panels, the complete script
needs to be re-run after manually modifying the parameters \(\kappa\), \(\phi\) and \(g\_{max}\). We ran the model for \(\kappa\) values of {10, 30, 100, 300, 1000}
and \(\phi\) values of {100, 300, 1000,
3000, 10000}. The value of \(g\_{max}\)
was chosen such that the infection had burned out (i.e., virus particles
remain infectious only for one generation, hence once there are no new
infections there will be no further spread) by inspecting the plots
produced. Note that, for a better representation of the data, different
values were used for the cognate panels in **S2** and
**S3 Figs**, as viral spread is generally more rapid in the
insect cells due to a higher fraction of complete virus particles.

The parameter values set in this file correspond to the top left
panels in **S2** and **S3 Figs**. Note that
the random seed is reset to ensure the reproducibility of the generated
panels.

#### Function for predicting infection dynamics

We specify a function for letting a population composed of virus
particles replicate and spread in a population of cells. Importantly,
the simulation is setup such that the composition of virus particles can
vary in terms of the segments present in them. This function generates a
3-dimensional array, with the rows being individual sims, columns
representing time points, and in the z-dimension 1 is the fraction of
infected cells, 2 is the realized MOI, and 3 is the fraction of cells
that is infected due to co-infection (i.e. infected cells in which
infectious three-segmented virus particles did not contribute to
infection). Note that the realized MOI and fraction of cells infected by
co-infection are not considered, but are kept in the function in case
they are of interest to anyone. This functionality is used in the
sim.virus.2() function described later on. Note that, in this version of
the function, if some virus populations go extinct, this will not cause
problems.

```
sim.virus <- function(tot.sims, max.time, ini.inf, vir.prod, inf.prob, num.cells, vp.types) {
  
  # Array to store the data on the number of infected cells, MOI and the 
  # contribution of incomplete particles to infection.
  sim.data <- array(NA, dim = c(tot.sims, max.time, 3))

  # Loop for the simulations
  for(i in 1:tot.sims) {
    
      # Set the number of infected cells to the starting value, exit.flag to zero,
    # and send data for t = 1 to array.
      inf.prev = 0
      inf.now  = ini.inf
      exit.flag = 0
      time = 1
      sim.data[i,time,c(1:3)] = c(ini.inf, NA, NA)
            
      # If loop for running all generations. If the population goes extinct or all
      # cells become infected, in this new version enters a value for every
      # generation for the number of infected cells, ranging from zero to num.cells.
      for(z in 1:(max.time-1)) {
        
          # Time: this formulation is a bit strange and a holdover from an earlier
        # version, but has been kept to avoid other complications.
          time = z + 1
          
          # Draw the total number of virus particles invading each cell. First 
          # determine the mean number of invading virus particles. Set to zero once 
          # all cells are infected.
          mean.lambda = (inf.now*vir.prod*inv.prob)/(num.cells - inf.now - inf.prev)
          
        # Then draw the number of invading particles for each cell
        lambda.tot = rpois(n = (num.cells - inf.now - inf.prev), lambda = mean.lambda)   
          
        # Determine which cells have > 0 vp, so that you only work with these
        invaded = which(lambda.tot > 0)
          lambda.tot.inv = lambda.tot[invaded]
        num.invaded = length(lambda.tot.inv)
        
          # Nested loop drawing the virus particle types for each cell, and 
        # determining whether cells are infected or not.
        
          inf.new = 0
        comp.now = 0    
      
        # Nest this loop which determines the identities of infecting virus
        # particles, so that if lambda is zero the script does not crash (because 
        # size = 0  in sample(), is not allowed), but instead exits the while loop
        # as the population has gone extinct under assumptions made (1 time point
        # window for infecting new cells).
        if(num.invaded > 0) {
          for(j in 1:num.invaded) {
                    
              # Now draw the number of hits for each type of virus particle for this 
              # cell, but only return unique values since we don't care how many of 
              # each type of virus particle is present, only if it is present.
                all.lambdas = unique(sample(x = 1:8, size = lambda.tot.inv[j], 
                                   replace = TRUE, prob = vp.types[,4]))
                
                # Now determine if the cell is infected, and for infected cells 
            # determine whether complementation between virus particles with 
                # an incomplete  set of genome segments was necessary for infection.
                inv.now = rbind(vp.types[all.lambdas,])
                if(sum(inv.now[,1]) > 0 & sum(inv.now[,2]) > 0 & sum(inv.now[,3]) > 0) {
                    inf.new = inf.new + 1
                  if(length(which(all.lambdas == 8)) == 0) comp.now = comp.now + 1
              }
        
            } # end of j loop for this cell
        }   
                
        # Update numbers
        inf.prev = inf.prev + inf.now
        inf.now = inf.new
        tot.inf = inf.prev + inf.new
        
          # Send data to array. Complementation result (comp.res) is an addition
        # made since this can only be calculated if there are new infections.
        if(inf.new > 0) {
          comp.res = comp.now/inf.new 
        } else {
          mean.lambda = 0
          comp.res = 0
        } 
        sim.data[i,time,c(1:3)] = c(tot.inf, mean.lambda, comp.res)
          
    } # end of z loop
    
  } # end of i loop
  return(sim.data)
}
```

#### General conditions for simulations

We start by defining the general conditions for which we will run the
passaging. These parameters have been chosen to be similar to the cell
culture conditions, and at the same time, representative for a situation
in which a virus population is expanding locally (i.e. the total number
of cells is kept small, so that the MOI increases locally as the
infection progresses). This situation is representative of a virus
replication and expansion in a mass of cells where the movement of virus
particles is constrained, as could occur in real life tissues or for
example in a plaque assay *in vitro*. A description of the models
is provided below, as well as the variable names used in the main
manuscript and other documentation (\(g\_{max}\), \(i\_0\), \(\phi\), \(\kappa\) and \(\rho\)).

```
# General parameters for the simulations that need to be set.
tot.sims  <- 1000   # The total number of simulations to be run for each scenario
max.time  <- 3    # Housekeeping: a maximum number of time steps allowed. 
                  # Parameter called g_max in other documentation.

# The biological parameters that we will keep constant over all simulations here.
ini.inf   <- 1      # The number of cells initially infected. Parameter called i_0
                  # in other documentation.
vir.prod  <- 10000  # The number of virus particles produced per infected cell.
                  # Parameter called phi in other documentation.
num.cells <- 10   # The total number of cells. Parameter called kappa
                  # elsewhere.
inv.prob  <- 10^-1.4    # The probability that each virus particle "invades" a new
                      # cell (i.e. that it enters the cell and can contribute 
                      # to infection if all genome segments are present).
                      # Parameter called rho in other documentation.

# Finally, set random seed for reproducibility.
set.seed(666)
```

#### Predictions in mammalian cells

Here we make predictions for the mammalian cells.

##### Random packager with co-infection by incomplete virus particles

Next, we need to specify what the virus particles look like with
respect to the packaging of viral genome segments into virus particles.
Based on empirical data we have a good idea of what this distribution
looks like for the real virus, so we generate a matrix containing this
information. Note that this matrix will also determine infectivity, so
it also has bearing on whether incomplete virus particles are infectious
(i.e. for simplicity, the generation of incomplete virus particles is
set to zero for generating a prediction in which incomplete particles do
not contribute to infection spread). First we consider the situation in
which we have a random packager in mammalian cells, and that incomplete
virus particles can complement each other and thereby cause cellular
infection.

```
# The first three columns indicate the presence or absence of a segment, and the
# final column represents the frequency at which that combination is present.
vp.types = array(dim = c(8, 4), data = c(
    0, 1, 0, 0, 1, 0, 1, 1,
    0, 0, 1, 0, 1, 1, 0, 1,
    0, 0, 0, 1, 0, 1, 1, 1,
    0.5, rep(0.09, 3), rep(0.06, 3), 0.05))
colnames(vp.types) <- c("Segment 1", "Segment 2", "Segment 3", "Frequency of VP type")

print(vp.types)
```

```
##      Segment 1 Segment 2 Segment 3 Frequency of VP type
## [1,]         0         0         0                 0.50
## [2,]         1         0         0                 0.09
## [3,]         0         1         0                 0.09
## [4,]         0         0         1                 0.09
## [5,]         1         1         0                 0.06
## [6,]         0         1         1                 0.06
## [7,]         1         0         1                 0.06
## [8,]         1         1         1                 0.05
```

```
# Rename the vp.types array for the random packager, to make it available later 
# on for normalization of the number of virus particles for the selective
# packager.
vp.types.rp = vp.types
```

Now we run the simulations for these conditions, and then first make
a plot of the mean, and then of individual replicates.

```
# Run the simulations using the simulation function
sim.data <- sim.virus(tot.sims = tot.sims, max.time = max.time, ini.inf = ini.inf, 
          vir.prod = vir.prod, inf.prob = inf.prob, num.cells = num.cells,
          vp.types = vp.types)

# Now determine the mean number of infected cells
mean.inf = rep(NA, max.time)
for(i in 1:max.time) mean.inf[i] = mean(sim.data[,i,1])

# Rename key outputs so they are still available if the same code is used to 
# explore other conditions further on.
sim.data.1 = sim.data
mean.inf.1 = mean.inf
```

##### Random packager without co-infection by incomplete virus particles

Next, we consider a random packager in mammalian cells, where
incomplete virus particles cannot complement each other and therefore do
not cause cellular infection. To this end, we modify the matrix for
virus particle composition, setting the frequency of all incomplete
variants to zero, and instead replacing them with empty particles.

```
# The first three columns indicate the presence or absence of a segment, and the
# final column represents the frequency at which that combination is present.
vp.types = array(dim = c(8, 4), data = c(
    0, 1, 0, 0, 1, 0, 1, 1,
    0, 0, 1, 0, 1, 1, 0, 1,
    0, 0, 0, 1, 0, 1, 1, 1,
    0.95, rep(0, 6), 0.05))
colnames(vp.types) <- c("Segment 1", "Segment 2", "Segment 3", "Frequency of VP type")

print(vp.types)
```

```
##      Segment 1 Segment 2 Segment 3 Frequency of VP type
## [1,]         0         0         0                 0.95
## [2,]         1         0         0                 0.00
## [3,]         0         1         0                 0.00
## [4,]         0         0         1                 0.00
## [5,]         1         1         0                 0.00
## [6,]         0         1         1                 0.00
## [7,]         1         0         1                 0.00
## [8,]         1         1         1                 0.05
```

Now we run the simulations for these conditions, and then first make
a plot of the mean, and then of some individual replicates.

```
# Run the simulations using the simulation function
sim.data <- sim.virus(tot.sims = tot.sims, max.time = max.time, ini.inf = ini.inf, 
          vir.prod = vir.prod, inf.prob = inf.prob, num.cells = num.cells,
          vp.types = vp.types)

# Now determine the mean number of infected cells
mean.inf = rep(NA, max.time)
for(i in 1:max.time) mean.inf[i] = mean(sim.data[,i,1])

# Rename key outputs so they are still available if the same code is used to 
# explore other conditions further on.
sim.data.2 = sim.data
mean.inf.2 = mean.inf
```

##### Selective packager

Finally, we consider a virus that perfectly packages all of its
segments into each virus particle produced. To do so, we only need to
specify that all segments are present in each virus particle in the
matrix for genome segment distributions. However, that would not lead to
a fair comparison: to produce the same number of complete virus
particles, the perfect packager can require considerably more genome
segments available for packaging compared to the random packager.
Therefore, we need to determine the number of genome segments available
to the random packager and then limit the total number of genome
segments available to the selective packager. For simplicity, the total
number of virus particles will be kept the same, and a fraction of empty
virus particles will be introduced. Note that this means that for this
virus, a sensible calculation of the MOI cannot be made with this
code.

```
# Determine the total number of genome segments packaged into virus particles
# for the random packager.
vp.segs = rep(NA, 8)
for(i in 1:8) vp.segs[i] = sum(vp.types.rp[i,(1:3)])*vp.types.rp[i,4]

# For a selective packager, the same calculation would render a value of 3x1= 3 
# (3 segments times a frequency of 1), leading to the normalization factor:
norm.pp = sum(vp.segs)/3

# The first three columns indicate the presence or absence of a segment, and the
# final column represents the frequency at which that combination is present.
# Here we consider the effect of a limit to the number of genome segments 
# available, based on the random packager.
vp.types = array(dim = c(8, 4), data = c(
    0, 1, 0, 0, 1, 0, 1, 1,
    0, 0, 1, 0, 1, 1, 0, 1,
    0, 0, 0, 1, 0, 1, 1, 1,
    (1-norm.pp), rep(0, 6), norm.pp))
colnames(vp.types) <- c("Segment 1", "Segment 2", "Segment 3", "Frequency of VP type")
print(vp.types)
```

```
##      Segment 1 Segment 2 Segment 3 Frequency of VP type
## [1,]         0         0         0                 0.74
## [2,]         1         0         0                 0.00
## [3,]         0         1         0                 0.00
## [4,]         0         0         1                 0.00
## [5,]         1         1         0                 0.00
## [6,]         0         1         1                 0.00
## [7,]         1         0         1                 0.00
## [8,]         1         1         1                 0.26
```

Now we run the simulations for these conditions, and then first make
a plot of the mean, and then of some individual replicates.

```
# Run the simulations using the simulation function
sim.data <- sim.virus(tot.sims = tot.sims, max.time = max.time, ini.inf = ini.inf, 
          vir.prod = vir.prod, inf.prob = inf.prob, num.cells = num.cells,
          vp.types = vp.types)

# Now determine the mean number of infected cells
mean.inf = rep(NA, max.time)
for(i in 1:max.time) mean.inf[i] = mean(sim.data[,i,1])

# Rename key outputs so they are still available if the same code is used to 
# explore other conditions further on.
sim.data.3 = sim.data
mean.inf.3 = mean.inf
```

##### Plotting the data

We create a single empty plot to represent all the results for the
mammalian cells, for all three packaging and infection scenarios.

```
# Create an empty plot for showing all data
par(mar = c(5, 6, 4, 2))

plot(x = c(1, max.time), y = c(0, log10(num.cells)), type = "n",
     main = " ", xlab = " ", ylab = "",
     xlim = c(0,max.time), ylim = c(0, log10(num.cells)), 
     cex.lab = 1, cex.axis = 2,
     las = 1)


# Plot individual replicates
for (i in 1:20) lines(x = (0:(max.time-1)), y = log10(sim.data.1[i,,1]), 
                           lwd = 0.25, lty = 3, col = "plum")
for (i in 1:20) lines(x = (0:(max.time-1)), y = log10(sim.data.2[i,,1]), 
                           lwd = 0.25, lty = 3, col = "lightgreen")
for (i in 1:20) lines(x = (0:(max.time-1)), y = log10(sim.data.3[i,,1]), 
                           lwd = 0.25, lty = 3, col = "lightblue")

# Plot the means
lines(x = (0:(max.time-1)), y = log10(mean.inf.1), lwd = 4, lty = 1, 
      col = "purple4")
lines(x = (0:(max.time-1)), y = log10(mean.inf.2), lwd = 4, lty = 2, 
      col = "darkgreen")
lines(x = (0:(max.time-1)), y = log10(mean.inf.3), lwd = 4, lty = 3, 
      col = "darkblue")
```

In this plot, the solid lines represent the mean of simulations for
one condition, and the light dotted lines represent individual
simulations. The purple lines represent the random packager when
co-infection by incomplete particles is allowed, green lines represent
the random packager without co-infection by incomplete particles, and
the blue lines represent the selective packager. Note that the mean
level of infection over time is plotted for all populations (and not
only those in which all cells become infected, as in some previous
versions). Note that although the mean is based on a large number of
simulations (1000), only 20 individual simulations are plotted to keep
the figure clear.

#### Predictions in insect cells

Next, we make predictions for the insect cell line. We reproduce the
script from above, but alter the distribution of genome segments across
virus particles, again based on empirical data.

##### Random packager with co-infection by incomplete virus particles

First, we consider the situation in which we have a random packager
in insect cells, and that incomplete virus particles can complement each
other and thereby cause cellular infection.

```
# The first three columns indicate the presence or absence of a segment, and the
# final column represents the frequency at which that combination is present.
vp.types = array(dim = c(8, 4), data = c(
    0, 1, 0, 0, 1, 0, 1, 1,
    0, 0, 1, 0, 1, 1, 0, 1,
    0, 0, 0, 1, 0, 1, 1, 1,
    0.302, rep(0.07, 3), rep(0.096, 3), 0.2))
colnames(vp.types) <- c("Segment 1", "Segment 2", "Segment 3", "Frequency of VP type")

print(vp.types)
```

```
##      Segment 1 Segment 2 Segment 3 Frequency of VP type
## [1,]         0         0         0                0.302
## [2,]         1         0         0                0.070
## [3,]         0         1         0                0.070
## [4,]         0         0         1                0.070
## [5,]         1         1         0                0.096
## [6,]         0         1         1                0.096
## [7,]         1         0         1                0.096
## [8,]         1         1         1                0.200
```

```
# Rename the vp.types array for the random packager, to make it available later 
# on for normalization of the number of virus particles for the selective
# packager.
vp.types.rp = vp.types
```

Now we run the simulations for these conditions, and then first make
a plot of the mean, and then of some individual replicates.

```
# Run the simulations using the simulation function
sim.data <- sim.virus(tot.sims = tot.sims, max.time = max.time, ini.inf = ini.inf, 
          vir.prod = vir.prod, inf.prob = inf.prob, num.cells = num.cells,
          vp.types = vp.types)

# Now determine the mean number of infected cells
mean.inf = rep(NA, max.time)
for(i in 1:max.time) mean.inf[i] = mean(sim.data[,i,1])

# Rename key outputs so they are still available if the same code is used to 
# explore other conditions further on.
sim.data.4 = sim.data
mean.inf.4 = mean.inf
```

##### Random packager without co-infection by incomplete virus particles

Next, we consider a random packager in insect cells, and that
incomplete virus particles cannot complement each other and therefore do
not cause cellular infection. To this end, we modify the matrix for
virus particle composition, setting the frequency of all incomplete
variants to zero, and instead replacing them with empty particles.

```
# The first three columns indicate the presence or absence of a segment, and the
# final column represents the frequency at which that combination is present.
vp.types = array(dim = c(8, 4), data = c(
    0, 1, 0, 0, 1, 0, 1, 1,
    0, 0, 1, 0, 1, 1, 0, 1,
    0, 0, 0, 1, 0, 1, 1, 1,
    0.8, rep(0, 6), 0.2))
colnames(vp.types) <- c("Segment 1", "Segment 2", "Segment 3", "Frequency of VP type")

print(vp.types)
```

```
##      Segment 1 Segment 2 Segment 3 Frequency of VP type
## [1,]         0         0         0                  0.8
## [2,]         1         0         0                  0.0
## [3,]         0         1         0                  0.0
## [4,]         0         0         1                  0.0
## [5,]         1         1         0                  0.0
## [6,]         0         1         1                  0.0
## [7,]         1         0         1                  0.0
## [8,]         1         1         1                  0.2
```

Now we run the simulations for these conditions, and then first make
a plot of the mean, and then of some individual replicates.

```
# Run the simulations using the simulation function
sim.data <- sim.virus(tot.sims = tot.sims, max.time = max.time, ini.inf = ini.inf, 
          vir.prod = vir.prod, inf.prob = inf.prob, num.cells = num.cells,
          vp.types = vp.types)

# Now determine the mean number of infected cells
mean.inf = rep(NA, max.time)
for(i in 1:max.time) mean.inf[i] = mean(sim.data[,i,1])

# Rename key outputs so they are still available if the same code is used to 
# explore other conditions further on.
sim.data.5 = sim.data
mean.inf.5 = mean.inf
```

##### Selective packager

Finally, we consider a virus that perfectly packages all of its
segments into each virus particle produced. To do so, we only need to
specify that all segments are present in each virus particle in the
matrix for genome segment distributions.

```
# Determine the total number of genome segments packaged into virus particles
# for the random packager.

vp.segs = rep(NA, 8)
for(i in 1:8) vp.segs[i] = sum(vp.types.rp[i,(1:3)])*vp.types.rp[i,4]

# For a perfect packager, the same calculation would render a value of 3x1= 3 
# (3 segments times a frequency of 1), leading to the normalization factor:
norm.pp = sum(vp.segs)/3

# The first three columns indicate the presence or absence of a segment, and the
# final column represents the frequency at which that combination is present.
# Here we consider the effect of a limit to the number of genome segments 
# available, based on the random packager.
vp.types = array(dim = c(8, 4), data = c(
    0, 1, 0, 0, 1, 0, 1, 1,
    0, 0, 1, 0, 1, 1, 0, 1,
    0, 0, 0, 1, 0, 1, 1, 1,
    (1-norm.pp), rep(0, 6), norm.pp))
colnames(vp.types) <- c("Segment 1", "Segment 2", "Segment 3", "Frequency of VP type")
print(vp.types)
```

```
##      Segment 1 Segment 2 Segment 3 Frequency of VP type
## [1,]         0         0         0                0.538
## [2,]         1         0         0                0.000
## [3,]         0         1         0                0.000
## [4,]         0         0         1                0.000
## [5,]         1         1         0                0.000
## [6,]         0         1         1                0.000
## [7,]         1         0         1                0.000
## [8,]         1         1         1                0.462
```

Now we run the simulations for these conditions, and then first make
a plot of the mean, and then of some individual replicates.

```
# Run the simulations using the simulation function
sim.data <- sim.virus(tot.sims = tot.sims, max.time = max.time, ini.inf = ini.inf, 
          vir.prod = vir.prod, inf.prob = inf.prob, num.cells = num.cells,
          vp.types = vp.types)

# Now determine the mean number of infected cells
mean.inf = rep(NA, max.time)
for(i in 1:max.time) mean.inf[i] = mean(sim.data[,i,1])

# Rename key outputs so they are still available if the same code is used to 
# explore other conditions further on.
sim.data.6 = sim.data
mean.inf.6 = mean.inf
```

##### Plotting the data

We create a single empty plot to represent all the results for the
insect cells, for all three packaging and infection scenarios.

```
# Create an empty plot for showing all data
par(mar = c(5, 6, 4, 2))

plot(x = c(1, max.time), y = c(0, log10(num.cells)), type = "n",
     main = " ", xlab = " ", ylab = "",
     xlim = c(0,max.time), ylim = c(0, log10(num.cells)), 
     cex.lab = 1, cex.axis = 2,
     las = 1)

# Plot individual replicates
for (i in 1:20) lines(x = (0:(max.time-1)), y = log10(sim.data.4[i,,1]), 
                           lwd = 0.25, lty = 3, col = "plum")
for (i in 1:20) lines(x = (0:(max.time-1)), y = log10(sim.data.5[i,,1]), 
                           lwd = 0.25, lty = 3, col = "lightgreen")
for (i in 1:20) lines(x = (0:(max.time-1)), y = log10(sim.data.6[i,,1]), 
                           lwd = 0.25, lty = 3, col = "lightblue")

# Plot the means
lines(x = (0:(max.time-1)), y = log10(mean.inf.4), lwd = 4, lty = 1, 
      col = "purple4")
lines(x = (0:(max.time-1)), y = log10(mean.inf.5), lwd = 4, lty = 2, 
      col = "darkgreen")
lines(x = (0:(max.time-1)), y = log10(mean.inf.6), lwd = 4, lty = 3, 
      col = "darkblue")
```

In this plot, the solid lines represent the mean of simulations for
one condition, and the light dotted lines represent individual
simulations. The purple lines represent the random packager when
co-infection by incomplete particles is allowed, green lines represent
the random packager without co-infection by incomplete particles, and
the blue lines represent the selective packager. Note that the mean
level of infection over time is plotted for all populations (and not
only those in which all cells become infected, as in some previous
versions). Note that although the mean is based on a large number of
simulations (1000), only 20 individual simulations are plotted to keep
the figure clear.
