# Supplementary material for: Incomplete bunyavirus particles can cooperatively support virus infection and spread
Source: PLoS Biol. 2022 Nov 15;20(11):e3001870. doi: 10.1371/journal.pbio.3001870 (PMC9665397; doi:10.1371/journal.pbio.3001870)
Supplement: Attachment — Submitted filename: Response to Reviewers.pdf [file pbio.3001870.s015.pdf]

Houtribweg 39, 8221 RA Lelystad, The Netherlands

26 August 2022

Response to reviewers (PBIOLOGY-D22-00778R1)

Dear Reviewers,

We thank you for your positive assessment, critical comments and constructive suggestions. In this letter, we provide a point-by-point response to all the comments you raised. We have taken onboard your feedback and think the new version of the manuscript has greatly improved. Below, reviewer comments are reproduced in italics and our responses in roman. In the revised manuscript, changes to the initially submitted version are highlighted in red.

We hope to have answered your questions and addressed your comments to your satisfaction, ultimately refining our manuscript to become acceptable for publication in PLOS Biology.

Please, note that the line numbers and reference numbers specified in our responses correspond to the line numbers and references of the revised manuscript.

Reviewer #1

General comments

This work addresses the question of whether distinct particles of a bunyavirus encapsidating an incomplete set of genome segments, so incomplete genetic information, can co-infect a cell and thereby reconstitute a complete genome and participate to within host spread and between host vector-transmission. This question is of high interest in Virology, definitely.

The experiments are of high standard and satisfactorily support some of the conclusions but not others. While the proof of concept that such bunyavirus particles can reconstitute full functional genomes upon co-infection in cell culture or in live mosquito vectors is well supported, that they do contribute to spread and transmission is not. Indeed, as discussed and illustrated through mathematical modelling, the MOI is key to this latter conclusion and the MOI is not empirically investigated in this paper. One key additional experiment would be to investigate MOI by using a mixture of green and red complete three segmented viruses and tracking the number of singly or doubly infected cells in infected mosquitoes, or after inoculation of a mammalian host or before and after transmission by mosquito. Another point that is key to this conclusion is what would happen if complete three-segmented particles co-exist with incomplete ones (reminiscent of natural situation)? would the incomplete particles contribute to spread and transmission. This is not tested in this paper. Such additional experiment would be required to conclude as in the title. In brief, that incomplete set of segments in "semi-infectious particles" can reconstitute a full genome through co-infection of cells is very convincing, but that these semi-infectious particles do so in more natural condition where they co-exist with complete ones, and so whether they do contribute to spread and transmission is not tested.

Another major drawback is more related to the writing and fairness in the presentation of the hypotheses/ideas enounced and tested here relative to the state of the art. This is utterly important as it determines the novelty of the finding and thus the scientific impact of this paper. In many instances, the ideas and hypotheses are presented as original and new, but these ideas have been proposed by others and predate this manuscript. Moreover, a fair amount of experimental work on incomplete particles produced in other viral systems have been published and amply discussed before. All this literature and input from others, the pre-existing relevant studies and discussions are not fairly acknowledged, and this is not acceptable.

Response: We thank the reviewer for the thorough analysis and assessment of the paper. We appreciate that the reviewer considers the question we are addressing in our manuscript of high interest in the field of virology and that we conducted high quality experiments to support most of our initial conclusions.

The main concern raised relates to the current manuscript title and its implications. We recognize that it will be appropriate to reword the title of the manuscript according to the evidence provided. We changed the title of the manuscript to “Incomplete bunyavirus particles can cooperatively support virus infection and spread”. This new title emphasizes the distinction pointed out by the reviewer: that bunyavirus incomplete particles can support infection and virus spread, whilst we cannot yet say with certainty to what extent they do so in practice. In addition, we have rephrased the original conclusion and made it more conservative. In the revised manuscript, we do not claim but imply, based on experimentation and mathematical modeling, that incomplete bunyavirus particles likely contribute to within-host virus spread and between-host transmission (lines 30-32, 94-97). To underscore this point, we emphasize that the presence of three-segmented virus in mosquito saliva upon infection with virus populations exclusively containing an incomplete set of genome segments strongly suggests that incomplete particles can contribute to between-host transmission. To quantify this contribution experimentally is currently not feasible, as incomplete particles are naturally present in the mix of particles that comprise bunyavirus populations.

The second major comment relates to the importance of the multiplicity of infection (MOI) for the success of complementation between incomplete particles. Perhaps it went unnoticed to reviewer 1, but we actually paid particular attention to this aspect in our study. Prior to illustrating the concept through mathematical modelling, we first investigated empirically the extent to which co-infection can occur *in vitro* at different MOIs by using the incomplete RVFV-SL particles encoding eGFP and mCherry2 (Fig. 2). Furthermore, we evaluated the success of complementation (in triplicate) in both mammalian and insect cells over a range of MOIs (from 0.001 to 0.25; included as Sup. Fig. 1). To highlight these results, we have decided to modify Fig. 4 by including a quantitative summary of these experiments in the main body of the manuscript (Fig. 4D).

The third point deals with the fact that complete three-segmented particles co-exist with incomplete particles in the natural context. We are aware that complete and incomplete particles co-exist in nature and we agree that studying the contribution of incomplete particles in the presence of complete particles would be desirable and highly relevant. However, we would like

to underline that investigating this in the laboratory is unfeasible at the moment, and will first require significant methodological advances. Bunyavirus infections naturally give rise to incomplete particle populations, and controlling the exact genomic content of specific populations, or purifying sub-populations with a specific genome content, is not yet possible.

Finally, discontentment is expressed regarding how we present some hypotheses/ideas and the insufficient citation of previous related work. We readily acknowledge that previous work has been both foundational and inspirational for the work we carried out here. However, we also would like to point out important differences between the present and previous work. A major difference between influenza A virus and Rift Valley fever virus (RVFV) is the selective versus non-selective genome packaging mechanisms, respectively. Producing only a very small proportion of complete infectious particles is a feature of bunyaviruses, and as such, it is conceivable that incomplete particles play a much more important role in the bunyavirus life cycle compared to other segmented viruses that were shown to use a selective genome packaging strategy. This drove our decision to mention influenza A virus work in the Discussion rather than in the Introduction section, and place more emphasis on a comparison with multipartite viruses. As a research team we also had considerable discussion on this aspect of the presentation, so we do understand reviewer 1's concern. We did not intend for our work to imply that incomplete particle complementation has not been conceptualized before outside of the bunyavirus field, and we apologize that this was unclear. To resolve this issue and clearly establish the primacy of other studies, we have now included brief mentions to previous works in the Introduction (lines 53-56) and Discussion (lines 435-437) sections.

Specific comments

Line 37: The Ref 1 is cited here to state the paradigm of packaging one of each segment in each virus particles. However, this cited paper already coin the idea that segmented viruses with non- or poorly selective packaging could be similar to multipartite viruses in some aspect and particularly that treated in this paper. Thus, the ideas pre-exist and should be acknowledged

Response: Our sole intention by citing reference 1 in the introductory sentence is to include a clear definition of what is accepted as the classical paradigm in relation to segmented and multipartite viruses, and to refer readers to an excellent review describing the context in which we place our work. We acknowledge that in reference 1, the authors already present an alternative view that challenges the classical paradigm. To avoid giving the impression that the

hypothesis coined as an alternative view is entirely new and original, we now cite reference 1 when we introduce this concept (lines 57-58).

Line 48: The references cited in the first part of the Introduction do not follow the "traditional view" as suggested here. On the opposite, they contributed to the development of the alternative view presented as original in this paper. Some of these references even explicitly confronted the classical against the alternative view. This is also the case in an important literature on Influenza virus A addressing "semi-infectious particles" (failing to express one or more segments) and their putative biological function, the complementation upon co-infection of cells and its role in reassortment. Such relevant predating work is totally ignored here.

Response: For clarity, we present the traditional distinction made between segmented and multipartite viruses. It was not our intention to present references 1-5 as work categorized as exclusively following the "traditional view". Simply, we think references 1-5 are excellent manuscripts to contextualize the type of work we were about to report. We are building knowledge on top of the concepts presented in those manuscripts. To acknowledge that, indeed, these works introduced an "alternative view", we are now also citing reference 1 (Michalakis & Blanc, *Annual Review of Virology* 2020) together with references 18 and 19 (Diefenbacher, Sun & Brooke, *Current Opinion in Virology* 2018; Jacobs *et al*., *Nature Communications 2019*) (lines 57-58), in the passage where we present the alternative hypothesis.

Regarding the important literature on influenza A virus semi-infectious particles, we recognize that we did not cite it in our original submission, not purposedly, but to focus more on viruses that have been less studied. We would like to emphasize the important difference between the selective versus non-selective genome packaging process in influenza A virus compared to RVFV. In addition, it should be noted that influenza A virus semi-infectious particles raise predominantly from a failure in expressing one or more genome segments, whereas for RVFV, whole genome segments are not being packaged. To recognize the important preceding work on influenza A virus, in the revised manuscript we have now introduced these manuscripts and placed them into context in relation to our work (lines 53-56; references Brooke *et* al., *Journal of Virology* 2013; Brooke, *Journal of Virology* 2017; Nakatsu *et al*., *mBio* 2016; Schelker *et al*., *PLoS Computational Biology* 2016; Russel, Trapnell & Bloom, *eLife* 2018).

Lines 54-59: all references showing that Influenza virus do not always package one copy of each of the 8 segments are omitted; it is very unfortunate because it is highly relevant and demonstrate that similar questions have been addressed before by others, in fact over the last ten years.

Response: We acknowledge this point of the reviewer and have now included an appropriate reference (line 55-56, Nakatsu *et al*., *mBio* 2016) referring to previous studies on genome packaging of influenza A virus. It is however worth noting that a major difference exists between the selective genome packaging process employed by influenza A virus, facilitated by the formation of a supramolecular RNP complex, compared to the non-selective process employed by bunyaviruses, characterized by the apparent absence of a supramolecular RNP complex, which may, at least in theory, make the dependence on genomic complementation by incomplete particles more important for bunyaviruses.

Lines 68-73: it is stated that segment packaging is poorly controlled in two bunyaviruses and yet they do propagate within and between hosts. Are the two references cited (REFs 14 and 15) showing this incomplete encapsidation in virions from host individuals or from insect vectors or is it from virions produced in cell culture. This would make a considerable difference, would call for caution and should be indicated.

Response: The two references cited (previously references 14 and 15; now references 21 and 22) refer to results obtained from *in vitro* studies. We agree with the reviewer that this detail must be clearly indicated, and we have now modified the statement accordingly (line 79).

Line 74: how would one know that the spread of RVFV and SBV is unaffected by non-selective packaging. They do spread, but whether this spread is affected or not is a different point? is this demonstrated somewhere?

Response: We agree with the reviewer's point, as indeed, we do not know if the spread of RVFV and SBV is affected or not by non-selective genome packaging. Although there is no direct evidence to support this idea, we believe it is reasonable to suggest so. Since RVFV and SBV can successfully sustain their dual life cycles between mammals and arthropods, possibly there are mechanisms to bypass the theoretical fitness cost of having a non-selective genome packaging process. In order to more clearly state that we refer to this situation in the context of a hypothesis, we rephrased our sentence (lines 80-82).

Line 87-88: This conclusion is overstated. The demonstration that incomplete particles contribute to spread and transmission is not presented. When impossible otherwise, two complementary incomplete virions can co-infect and reconstitute complete segment sets that initiate infection. But no information is given on whether and how often this occurs in the presence of virions containing a complete segment set.

Response: The discussion about providing insufficient evidence for the original conclusion has already been accepted and addressed above. As a result, we have modified the title (lines 1-2) and conclusion (lines 30-32, 94-97) accordingly.

Results

Line 110-113: please explain why the fast spread of three segmented viruses impedes the accurate assessment of co-infection rate.

Response: When assessing the probability of co-infection, we sought for an assay that enabled us to quantify accurately to what extent cells are susceptible to more than one virus particle after an initial exposure to a certain multiplicity of infection. Knowing that iRVFV-SL particles are unable to spread, an assay in which we determine the fraction of co-infected cells after exposure to two different populations of iRVFV-SL was ideal, as we will only look at a single cycle of infection. On the contrary, three-segmented bunyaviruses can infect, replicate and spread further with ease. After a simultaneous infection with two fluorescently marked three-segmented viruses, a fraction of the cell population will also become co-infected. However, since these viruses will rapidly generate virus progeny and possibly even reassortant viruses, new cells will become co-infected upon multiple cycles of infection, complicating an accurate assessment to what extent cells are susceptible to co-infection. In the first case, a cell co-expressing the two fluorescent proteins will only be the result of an infection with two iRVFV-SL particles, whereas in the second case, it could be the result of an infection with a single particle containing two different S segments (not necessarily a co-infection). To clarify this further, we have added a short explanation in lines 120 and 123-125.

Lines 163-166: Are the batches of rescued iRVFV-ML particles purified virions? Is the presence and integrity of such virus particle verified in this specific case? Figure 3 does present virus particles with M and L only but it is a drawing. It would be nice to show electron microscopy images of these particles.

Response: The batches of iRVFV-ML particles were rescued after transfection and concentrated by ultracentrifugation as described in lines 516-518. In Fig. 3 we show that cells exposed to iRVFV-ML do not show any expression of viral proteins and that the S genome segment could not be detected with a segment-specific RT-qPCR. Fig. 4B-C also confirms the absence of viral protein expression, while in Fig. 5B no replication of the viral genome is taking place. Absence of viral genome replication results from the absence of nucleocapsid protein encoded by the S segment, which is an essential viral replication machinery factor. Finally, in Fig. 5C we directly visualize that iRVFV-ML particles only contain the M and L genome segments and lack the S segment. It is worth noting that an electron microscopy analysis of the samples will not prove or disprove the genomic composition of iRVFV-ML particles, as the number and identity of the ribonucleoproteins inside virions cannot be identified using this method.

Figure 4b, why don't we see rare signal for the Gn in cells incubated with iRVFV-ML alone. Some rare cells should express this Gn protein even if the infection does not propagate (as is the case for cells incubated with iRVFV-SL-eGFP alone where few cells are singly infected and produced GFP).

Response: The S and L segments of RVFV encode the viral machinery essential for genome replication and transcription (i.e. the nucleocapsid protein and an RNA-dependent RNA polymerase, respectively). The glycoproteins Gn and Gc, essential for viral entry and assembly of progeny virions are encoded by the M segment. Therefore, iRVFV-SL-eGFP particles have replicative capacity but cannot spread. On the other hand, iRVFV-ML particles, lacking the S segment and thus nucleocapsid expression, cannot replicate or spread. Consequently, it was expected that single cells exclusively infected with iRVFV-SL-eGFP particles express eGFP, but that cells infected with iRVFV-ML particles do not show expression of any viral protein. An explanation about the non-spreading nature of iRVFV-SL-eGFP particles and non-replicating nature of iRVFV-ML particles was initially given in lines 129-134 and 170-174.

Lines 221-223: this is in cell culture and not in vivo in mammals

Response: As originally stated in lines 221-223 from the initially submitted manuscript (now lines 237-239), we clearly mention that since incomplete particles were able to generate a productive infection *in vitro*, we hypothesized that incomplete particles can also play a role in between-host virus transmission and proceed to describe our *in vivo* findings from the mosquito experiment.

Lines 244-246: would it be possible to try to mosquito-transmit this potentially reconstituted three-segment virus to a mammal host and see if infection is efficient? This would be extremely valuable as it would demonstrate the transmission that is here solely speculative.

Response: An experiment confirming that mosquitoes can transmit the reconstituted three-segmented virus to a mammalian host would be interesting to perform, however, the execution of such experiment is very complex and currently not feasible. Noteworthy, bypassing the barrier of co-infecting the mosquito midgut with a mixture of incomplete particles, reconstituting an infectious virus able to disseminate up to the mosquito salivary glands, as shown in our study, constitutes the major bottleneck in the between-host transmission cycle. Therefore, we cannot think of any reason to believe that the reconstituted virus recovered from mosquito saliva would not be transmissible to a mammalian host. To acknowledge that additional experiments would be valuable, we have included this suggestion (lines 417-420).

Lines 252-278 and *Figure 7a: Isn't this model trivial in the sense that the outcome could not be different, owing to the parameters of the model?*

Response: We think of mathematical modeling as a useful scientific tool to (i) test whether proposed mechanisms lead to a particular outcome or (ii) predict the behavior of a system, in particular when it is not feasible to do so experimentally. In this case, we were interested in predicting the contribution of incomplete particles to spread in a natural bunyavirus population (composed of both incomplete and complete particles). Given that it is not (yet) possible to do so experimentally, we used a modeling approach to compare three different scenarios. As already documented in the Supplementary Information (and contrary to the reviewer's assertion), we decided *a priori* on a scenario to model and on reasonable model parameter values. We generated and presented quantitative predictions based on what we thought was the most representative scenario.

Despite the fact that these model parameters were chosen *a priori* and independent of model predictions, it is still relevant to consider to what extent they have an effect on the predictions. We have therefore performed a simple sensitivity analysis, considering what happens when the two key model parameter values are varied. The result is briefly mentioned in the Results section (lines 303-312) and explained in detail in Supplementary File 1 and Supplementary Figs. 3 and 4. Overall, these results confirm that the patterns observed in Fig. 7A are not dependent on the

exact model parameters chosen: incomplete particles increase the rate of virus spread under most scenarios.

As we performed the sensitivity analysis, we noted that it was best to plot the mean number of infected cells calculated over all the simulated populations. Initially, we had plotted only the subset of populations in which the virus had not gone extinct and in which not all cells had become infected yet. This approach fails to capture the dynamics well when many populations go extinct and can amplify the effect of having some stragglers with slow spreading infection. Consequently, the means plotted in Fig. 7A now look slightly different, but they better represent the patterns for the specific conditions chosen, as well as allowing a better comparison for other parameter values (Supplementary Figs. 3 and 4).

<u>Lines 280-298</u>: The model appears similarly trivial in that the outcome directly stem from the parameters of the model. In fact, the model is built for this outcome.
The result of these models is not in itself interesting (related to comments above). What would be interesting, and to my view crucial for the main conclusion of this paper (basically what is in the title), is an empirical estimate of the MOI. With the three-segmented green and red viruses, it appears possible to estimate the MOI in vivo by monitoring the frequency of non-infected cells, of singly-infected cells versus doubly-infected. This would allow to conclude that indeed co-infection by incomplete two-segmented particles can or cannot significantly contribute to viral spread within and between hosts. I am not totally sure how feasible it is on a technical ground but it would be a very important add up to the paper.

Response: We object to the unsubstantiated assertions the reviewer is making here: the models were generated to explore the behavior of this system over a wide range of conditions, as we cannot (yet) do so experimentally. In the previous modeling section, we used more complex models –with up to three free parameters– to generate infection dynamics. However, the model used to generate this prediction does not have any free parameters; it only requires the distribution of genome segments over virus particles to generate a prediction. We have used empirical values of this distribution for insect and mammalian cells, as well as 40 sets of randomly generated distributions to address the generality of these trends (see also Supplementary File 3). It is untenable to state that this model was built for any outcome.

Concerning empirical measurements of MOI in multicellular hosts, we think these types of studies are worthwhile scientific endeavors, but properly performing these experiments and modeling

would represent a whole other study. Moreover, studies on MOI that have been performed highlight that MOI is a dynamic property that depends on the exact conditions found, and typically tends to increase over time (at least in early infection). This reinforces our conclusion that incomplete particles may play a role in virus spread, at least at some points during the infection cycle.

Discussion

Line 304, *again all the relevant literature on influenza virus A is ignored. This is annoying because it does not give credit to those scientists that had the same idea and/or addressed similar or related question before. The feeling that this type of omissions gives to the reader is that the authors are trying to oversell the results. As an illustration, line 305 says "we hypothesized" this is not true, others hypothesized and tested before and they must be acknowledged. Again, Line 307, that non-specific packaging of segmented genomes may poses questions similar to those that have emerged from the biology of multipartite viruses has been written before by others.*

Response: We have already agreed on including more literature related to articles addressing similar questions using influenza A virus (lines 53-56). However, it should be noted that in our statements we explicitly refer to testing this hypothesis for bunyaviruses, which contrary to influenza A virus, do not selectively package their genome segments. As explained above, we find this difference between selective (influenza A virus) versus non-selective (RVFV) genome packaging to be of particular importance in the context of our paper. Finally, we also would like to note that, to our knowledge, this type of evidence has not been shown before for a bunyavirus, and that is exactly what we are referring to.

Figure 8: *This figure may not be necessary it does not provide additional information and just summarizes the model which is already quite clear along the text.*

Response: We are very glad to know that the ideas and results we convene in our work were presented sufficiently clear in the text, but we would argue that Fig. 8 provides a good graphical summary that can be of great help for the reader to recapitulate our findings in a simple way. Thus, we think the readers will benefit from keeping the figure in the manuscript.

Methods

Line 468: *RT-qPCR determines a concentration of RNA or a copy number but in no way a viral titer. The absence of N protein may impact on infectivity of particles for example.*

Response: Indeed, RT-qPCR allows the determination of RNA concentration or copy number, and whenever possible, another test should be used to determine viral titers. For three-segmented RVFV and incomplete RVFV-SL particles that have replicative capacity, we determined virus titers using an end-point dilution assay in combination with either an immunoperoxidase monolayer assay or direct microscopy detection of fluorescent protein expression (lines 527-534 and 603-624). However, as initially stated in the manuscript, the conventional titration assay is not applicable to determine the titer of non-replicable incomplete RVFV-ML particles. Consequently, we made a titer estimation, and stated it in those terms, by quantifying the RNA genome copies and comparing similar measurements of RNA copies of virus stocks with known infectious titers (lines 174-178 and 545-550).

Reviewer #2: This paper by Bermúdez-Méndez et al examines the potential for incomplete bunyavirus particles to contribute to replication through complementation. They use a nice set of recombinant viruses missing individual segments to explore the potential for complementation through co-infection. Critically, they demonstrate in mosquitos that a blood meal containing only a mix of incomplete particles can result in replication and dissemination of replication competent virus to the salivary glands, an important step in transmission. They complement their experimental work with modeling-based analyses aimed at quantifying the contribution of incomplete particles to infectious virus production in different host environments and across a range of MOIs and genome packaging probabilities.

Altogether the work is very nicely done and significantly extends our understanding of bunyavirus population dynamics. The paper also has broader significance in contributing to a larger body of work exploring fundamental questions concerning the lifestyles of segmented and multipartite viruses. The paper is clearly written, and the conclusions are generally well supported by the data. I have only a few minor comments:

Response: We thank the reviewer for the comprehensive review of the manuscript and highly appreciate the very positive comments regarding the presented work. We are very glad that besides providing new evidence for a concept of great interest in virology, our work is placed in the broader context of the intersection between the life cycles of segmented and multipartite viruses.

Lines 54-57: The authors need to mention two prominent examples of other segmented viruses that produce large numbers of incomplete particles: influenza viruses (PMID: 23283949) and jengminviruses (PMID: 27569558). This is important to make clear the phenomenon described here is not unique to bunyaviruses.

Response: We agree with the reviewer. To improve the presentation of the available knowledge, we have included a passage (lines 55-56, 63-66) in the Introduction to refer explicitly to other segmented viruses that also produce incomplete particles during infection.

Line 143: The "predictive model" used should be described more extensively in the main text so that readers don't have to dig into the supplement to understand what exactly was done and how to interpret the results. This is true for the modeling analysis later in the paper.

Response: We acknowledge the point raised by the reviewer. To improve the clarity of the modeling sections, we have introduced more detailed explanations about the rationale behind all the models included in the main body of the manuscript (lines 714-824).

Fig 5B,C: This figure would benefit from presenting some quantification of these data rather than simply representative images

Response: We understand the interest of the reviewer to see quantitative data from the microscopy images, however, the only purpose of the smFISH analysis in this case was to confirm the presence or absence of a particular viral genome segment within an infected cell or a mature virion to verify the genomic composition of the recombinant viruses generated by reverse genetics. In fact, counting data on images of Fig. 5B-C would not change our conclusion that the M segment is lacking in iRVFV-SL particles and that the S segment is lacking in iRVFV-ML particles.

Line 363-4: The authors need to be careful to say this suggests incomplete particles could potentially contribute to transmission, as it does not yet suggest they play a significant role in transmission as the authors state.

Response: As explained above in our response to reviewer 1, we do not yet have direct evidence for a significant contribution of incomplete particles to between-host virus transmission. However, taking together the facts that RVFV produces a large number of incomplete particles and that these particles can complement each other to surpass the mosquito midgut barrier and disseminate to the mosquito salivary glands even in the initial absence of complete particles, we propose that incomplete particles may play a role in transmission. Together with our experimental findings, our mathematical models suggest a potential contribution of incomplete particles to virus spread, likely serving as a strategy of bunyaviruses to establish productive infections. We have slightly rephrased this idea in lines 30-32, 94-97 and 452-467.

Reviewer #3: *this paper is an excellent piece of scientific research. However I have a couple of things that I think the authors need to consider. However, I must commend the authors for an elegant piece of work with clear descriptions and outcomes*

Response: We are grateful for the positive evaluation of the reviewer.

Firstly, you are using defective cells in terms of the interferon response and the mosquito cells lacking an RNAi response, the mosquito is less problematic as you do the in vivo mosquito infections. However, this should be mentioned.

Response: We acknowledge the point of the reviewer. To be entirely clear about the condition of the cell lines used in our experiments, we introduced a few clarifying sentences (lines 386-389).

Please justify the use of saliva and the bodies. It would also be interesting to see if you could identify whether these complemented viruses were able to readily overcome the midgut barrier.

Response: The analysis of mosquito bodies and saliva was used to determine virus infection and dissemination in the mosquito, respectively. We indeed isolated infectious virus from the bodies and saliva of several mosquitoes that were fed with a bloodmeal exclusively containing incomplete virus particles (Fig. 6). Importantly, virus isolation from mosquito saliva confirmed that upon co-infection of midgut cells, the reconstituted virus was able to disseminate to the mosquito salivary glands, overcoming the major bottleneck in between-host transmission. Although interesting, the extent and easiness with which the reconstituted virus overcame the midgut barrier remained out of the scope of this manuscript, but we have no reason to believe that the reconstituted virus is different from an authentic three-segmented virus.

Also during the mosquito infection in the wild, what is the titre of the infection? If so is it likely that there are co-infections of cells in the midgut at those low MOI. This should also be considered in the discussion.

Response: Based on previous knowledge obtained from experimental RVFV transmission studies from lambs to mosquitoes (Wichgers Schreur *et al.*, *npj Vaccines* 2020), it can be deduced that for a mosquito to become infected after having a blood meal from a ruminant (e.g. lamb), the animal has to be in the peak viremia phase, at which virus titers are typically in the range of 10^5-10^7 $TCID_{50}$/mL. Therefore, we consider the 10^{7}-10^8 $TCID_{50}$/mL virus titers of the blood meals

used in our mosquito experiment to be comparable to the peak viremia of an infected animal and to resemble the probability of co-infection of midgut cells similar to that of a natural situation. To address this point, we have introduced a brief explanation in the Discussion section (lines 396-399).

Minor comments

Line 429 = please either cite a paper where the methods used are described or briefly describe the methods.

Response: The plasmids and transfection conditions used to generate the recombinant viruses using reverse genetics are described in lines 488-501 and 505-514. We also refer to a paper where the method was used (Kortekaas *et al*., *Journal of Virology* 2011) in lines 490 and 507.

Reviewer #4: Bermudez-Mendez and colleagues present a study of the role of incomplete virus particles in rift valley fever virus replication and transmission. Their overall hypothesis is that bunyaviruses, due to non-selective packaging, produce many virus particles that lack one or more of the three segments required for successful completion of the virus life cycle. This study is inherently interesting as it addresses a fundamental question in virology pertaining to the role of so-called defective virus particles: can apparently defective viruses contribute to transmission, pathogenesis, etc., and under what circumstances. In addition, the virus chosen to address this question is timely since it is of significant concern as an emerging animal and human pathogen. This will be of interest to readers of PLoS biology with an interest in virology, emerging viruses and virus population biology.

To address their questions, the authors generate several RVFV constructs that express markers and/or lack one of the three segments. These were then applied to cells at different MOIs and coinfection monitored by several relevant methods, including some clever imaging techniques. Results from empirical studies were compared with models that attempted to derive theoretical estimates for the likelihood of coinfection under various conditions. Incomplete particles were also fed to mosquitoes and infection in mosquito bodies and saliva measured.

Collectively, the results of the studies demonstrate quite convincingly that RVFV particles lacking a full genome can complement others lacking a different segment, and themselves be complemented in vitro and in vivo. This is the core finding reported in this manuscript. It is generally well written and clear and as I mentioned above, will undoubtedly be of interest to this journal's readership.

Response: We thank the reviewer for the very positive assessment of the manuscript and highly appreciate the recognition of the importance of the question we addressed and that the paper is indeed of interest to a broad readership.

I have three concerns to raise about the manuscript in its current form for the authors' consideration.

1) *In vivo studies. The work with mosquitoes is key to this paper because it provides biological relevance. For too long, studies of this type were conducted exclusively in tissue culture, which has led to several persistent misconceptions in virology. I found it striking that no animal studies, analogous to those conducted with mosquitoes, were included. Mainly because the authors*

predict that the role of incomplete virus genomes ought to be greater mammals than arthropods. This lack of symmetry limits the scope of the paper to a fairly large degree.

Response: We agree with the reviewer on the biological relevance of the findings from our mosquito experiment, and we also agree on the value of an animal study analogous to the one we conducted with mosquitoes. Since performing such an experiment was unfortunately not feasible for us, we have included in the Discussion (lines 417-420) that evaluating if it is possible to rescue infectious virus *in vivo* from a mammal exclusively exposed to incomplete particle populations is an important matter to address in future research. Finally, we note that -based on our observations– the role of incomplete particles during between-host transmission is expected to be greatest during the transition from mammals to mosquitoes. We can expect this because the distribution of genome segments over virus particles in the inoculum will be determined by the host in which replication occurred, and there is a lower frequency of complete particles in mammalian cells than in insect cells. In that perspective, the infection of mosquitoes with an inoculum lacking complete particles is the most convincing demonstration that incomplete particles can contribute to transmission.

2) To a non-modeler, the modeling sections of this work seem poorly developed and integrated. While I can see from the figures that experimental and model-derived predictions are aligned, I can't tell how much weight to put on this. I also found that the results in figure seven weren't clearly connected to the rest of the paper. I think that much more could be done with this piece of the study. A more clear discussion of the results, what they mean, and how they related to the rest of the paper might be a place to start. As an adjunct to this, some helpful, simple comparisons such as between the predicted and observed (co-)infection probabilities of GFP and mCherry viruses at a given MOI could be very helpful. Can these models be extended to animal infection? I think that this part of the paper could be significantly better than it is.

Response: We understand the reviewer's concern. As our work focuses on the experimental results, we only provided a brief and intuitive description of the modeling methods and results in the main body of the paper. We have extended our description of the methods to provide more details in response to reviewer 2. Moreover, we have made additional changes in response to this specific comment. First, we now provide a summary of the main model selection results to show that the model depicted in Fig. 2G has solid empirical support (lines 154-157 and Supplementary Table 1), and have added some additional clarification on why this modeling result is relevant (lines 160-162). Second, we have better integrated the results presented in Fig. 7, providing a

clear rationale for using models to explore infection dynamics (lines 268-277 and 330-332) and what we learn from the modeling results (lines 314-318). Finally, we followed the reviewer's suggestion and carefully revised our discussion of the modeling results (lines 349-353 and 357-360).

3) While there is a lot left to do and I can imagine that there are many more papers coming from this very interesting project, I think it would be very helpful to include a better discussion of MOI. While any virologist will know what the authors mean, a clear definition should be provided. The reason for this is that, as the authors allude to in the text, the field is increasingly aware of the disconnect between MOI and virus genome copies, and the possibility that viruses may be transmitted as collective groups. It would be helpful to address this because if, for example, viruses aggregate or arrive within vesicles, the interpretation of the results would be slightly different (in interesting ways.)

Response: We thank the reviewer for this comment. Having extended the description of the models and their implications, we took the opportunity to emphasize more the role that MOI likely plays in this phenomena (lines 316-319). Regarding the possibility that viruses may be transmitted as collective infectious units, we had already considered it and commented on this idea in lines 425-427.
